# ﻿New species and newly recorded species of the family Strophariaceae (Agaricomycetes, Agaricales) in China

**DOI:** 10.3897/mycokeys.124.166503

**Published:** 2025-11-10

**Authors:** Chunyu Lei, Guiyin Deng, Jiahui Huang, Yalun Shen, Wenbo Yan, Enjing Tian, Yongping Fu

**Affiliations:** 1 Sanjiang Laboratory, Changchun, Jilin Province, 130018, China Sanjiang Laboratory Changchun China; 2 Engineering Research Center of Edible and Medicinal Fungi, Ministry of Education, Jilin Agricultural University, Changchun, 130118, China Jilin Agricultural University Changchun China

**Keywords:** *
Agrocybe
*, morphology, *
Pholiota
*, phylogeny, *
Pyrrhulomyces
*

## Abstract

Two new species, *Pholiota songjiangensis* and *Pyrrhulomyces pileocystidiatus*, and one species newly recorded in China, *Agrocybe eduardii*, are described in this study. All of these species belong to the family Strophariaceae. *Pholiota songjiangensis* is characterized by a pallid pileus with appressed and concentric squamules, a white stipe covered with light yellowish-brown small scales, ellipsoid to ovoid basidiospores with an obvious germ pore, pleurocystidia as chrysocystidia, and cheilocystidia with two shapes: elongate-cylindrical with a capitulate apex and narrowly lageniform. *Pyrrhulomyces pileocystidiatus* is characterized by a bright orange-red to ochraceous brown pileus with an obtuse umbo, bitter taste, blackening basidiomata, pleurocystidia as chrysocystidia, and broadly clavate and orange-red pileocystidia. The specimens of *Agrocybe eduardii* collected from China in the present study closely matched the original morphological description of this species. These three species are described and illustrated, and phylogenetic analysis of a multigene dataset (ITS+nrLSU) is presented. Morphological and phylogenetic analyses confirmed that *Pholiota songjiangensis* and *Pyrrhulomyces pileocystidiatus* were distinctly different from other *Pholiota* and *Pyrrhulomyces* species, respectively. The Chinese samples formed a monophyletic group with the holotype of *Agrocybe eduardii*, confirming its newly recorded status from China when combined with the morphological evidence. Keys to species of *Pyrrhulomyces*, *Agrocybe* from China, and Pholiota subgenus Pholiota from China are provided.

## ﻿Introduction

The family Strophariaceae Singer & A. H. Sm. (Agaricomycetes, Agaricales) was validated by Singer and Smith (1946). This family has a cosmopolitan distribution, and most genera are found in different parts of the world (Noordeloos 2011). In its original concept, Strophariaceae included nine genera from two subfamilies (Stropharioideae and Pholiotoideae), based on the color of the spore print (Singer 1963, 1986). Kühner (1980, 1984) presented a broader concept of this family than Singer, in which many dark-spored genera from Singer’s families, i.e., Coprinaceae, Bolbitiaceae, and Cortinariaceae, were included in Strophariaceae. Strophariaceae comprises 18 genera according to the 10^th^ edition of the Dictionary of Fungi (Kirk et al. 2008). The circumscription of Strophariaceae has changed substantially since the advent of molecular phylogeny. Many genera of this family have now been phylogenetically placed in various families, and some new genera have been added into Strophariaceae (Moncalvo et al. 2002; Jacobsson and Larsson 2007; Matheny et al. 2015; Kalichman et al. 2020; Tian and Matheny 2021). In our systematic taxonomic study on the family Strophariaceae, we discovered some new species and newly recorded species in China, mainly involving the following three genera: *Pholiota* (Fr.) P. Kumm, *Pyrrhulomyces* E.J. Tian & Matheny, and *Agrocybe* Fayod.

The genus *Pholiota* was first proposed as Trib. Pholiota by Fries in 1821, comprising 16 species, and later elevated to generic status by Kummer (Smith and Hesler 1968). The genus *Pholiota* is distributed worldwide, especially in the northern temperate zone, and currently contains approximately 150–160 species (Smith and Hesler 1968; Jacobsson 1991; Holec 2001; Kirk et al. 2008; Noordeloos 2011; Holec et al. 2014; He et al. 2019; Huang et al. 2024; Liu et al. 2024). Approximately 64 species of *Pholiota* have been reported in China, including 16 that belong to the subgenus Pholiota (Mao and Jiang 1993; Tian and Bau 2004; Bau et al. 2005; Tian and Bau 2011a, 2011b; Tian et al. 2016; Liu et al. 2024; Huang et al. 2024). Species of this genus grow on wood, sawdust, humus, soil, and, more rarely, on sphagnum beds or charcoal (Smith and Hesler 1968; Jacobsson 1991). Some species of this genus are edible and medicinal, such as *Ph. adiposa* (Batsch) P. Kumm. and *Ph. aurivella* (Batsch) P. Kumm. (Smith and Hesler 1968; Wu et al. 2019). However, this genus also includes mildly toxic species (Smith and Hesler 1968; Wu et al. 2019).

However, the classification system of *Pholiota* remains controversial. Different taxonomists proposed various viewpoints based on different concepts of this genus, ranging from the broad concept of 7 subgenera and 16 sections to the narrow concept of 3 subgenera and 8 sections (Singer 1963; Smith and Hesler 1968; Singer 1975; Jacobsson 1991; Holec 2001; Jacobsson 2012). Recent research has shown that the genus *Pholiota* remains polyphyletic, and *Pholiota* sensu stricto comprises at least two major subgenera: subgen. Pholiota and subgen. Flammuloides (Tian and Matheny 2021).

The genus *Pyrrhulomyces* was established by Tian and Matheny (2021) to accommodate *Pholiota astragalina* (Fr.) Singer, which was transferred from the genus *Pholiota* and recombined as *Pyrrhulomyces astragalinus* (Fr.) E.J. Tian & Matheny. The genus *Pyrrhulomyces* currently comprises only two species, *Pyrrhulomyces amariceps* E.J. Tian & Matheny and *Py. astragalinus*. This genus is characterized by a brightly colored pileus, blackening basidiomata with a bitter taste, smooth basidiospores without a germ pore under light microscopy, the presence of pleurochrysocystidia, a peculiar shape of the cheilocystidia, and association with the late stages of conifer wood decay. *Pyrrhulomyces* is similar to *Stropharia* and *Hypholoma* but differs from most species in these two genera in its brown spore deposit (not purplish brown), and the absence of an annulus, a germ pore in basidiospores, and acanthocytes in all tissues of the basidiocarp (Farr 1980; Singer 1986; Cortez and Silveira 2008).

The genus *Agrocybe* was established by Fayod in 1889, with *Agrocybe praecox* (Pers.) Fayod designated as the type species. Exhibiting a cosmopolitan distribution, *Agrocybe* primarily occurs in the temperate regions of Asia, Europe, and North America (Flynn and Miller 1990). Approximately 100–110 species have been recognized worldwide (He et al. 2019; Kiyashko et al. 2022; Li et al. 2023), 13 of which have been reported in China (Jin 2012; Jin and Bau 2012; Wang et al. 2018; Liu et al. 2021; Li et al. 2023). Species of *Agrocybe* typically grow on soil in forests or grasslands, although some are coprophilous, lignicolous, or plant-pathogenic. The characteristics of this genus are as follows: most species possess an annulus (occasionally absent); the stipe base usually bears white rhizomorphs; lamellae are adnexed to sinuate; a partial veil is present or evanescent; pleurocystidia are present in lamellae, or only cheilocystidia developed; the hyphae of trama are parallel to subparallel; basidiospores are ovoid, ellipsoid or fusiform, honey-yellow to pale brown, smooth-walled, and usually with a germ pore (occasionally indistinct or absent); pileipellis is a hymeniderm or ixohymsniderm, composed of globose, clavate, subglobose, pyriform, or vesiculose cells (Singer 1975; Watling 1982; Niveiro et al. 2020).

In this study, two new species, *Ph. songjiangensis* and *Py. pileocystidiatus*, and a species newly recorded in China, *A. eduardii*, were discovered based on morphological characteristics and phylogenetic analyses. These results are presented in the following sections.

## ﻿Materials and methods

### ﻿Morphological studies

The mushroom specimens were collected and photographed in the field. The specimens were then dried using a dryer and deposited in the Herbarium of Mycology at Jilin Agricultural University (HMJAU) in Changchun City, China.

Specimens were documented using color descriptions from Kornerup and Wanscher (1978). Microscopy slides were prepared using squash mounts and freehand sections. Sections were mounted in 5% KOH solution and observed under a light microscope. Congo Red solution was used as a stain when necessary to enhance visibility. Dextrinoid reactions of the spores were tested using Melzer’s reagent (Singer 1986). The microscopic structures were measured and examined following Tian and Matheny (2021) and Tian et al. (2021). Line drawings of microstructures were made from the rehydrated samples.

The following abbreviations are used in the morphological descriptions:

**L**: Number of full-length lamellae extending to the stipe.

**I**: Number of short lamellae (lamellae) between adjacent pairs of full-length lamellae.

**Q**: Basidiospore length-to-width ratio.

**Q_m_**: Mean Q value ± standard deviation across all studied specimens.

### ﻿DNA extraction, PCR, and data set assembly

Genomic DNA was extracted from specimens using a Plant Genomic DNA Kit (Beijing Solarbio Science & Technology Co., Ltd., China). The following gene regions were amplified and sequenced using the primer pairs ITS1/ITS4 (White et al. 1990; Gardes and Bruns 1993) for the ITS and LR0R/LR7 (Vilgalys and Hester 1990) for the nrLSU. PCR amplification was performed in 25 μL reaction volumes containing: 8.5 μL ddH_2_O, 12.5 μL 2 × PCR Master Mix, 1 μL each of forward and reverse primers, 2 μL DNA template solution. The resulting DNA sequences were deposited in the GenBank database. Following amplification and sequencing, the ITS and nLSU sequences were trimmed and concatenated using SeaView 5.1 and AliView 1.28 (Larsson 2014; for AliView). A combined ITS+nrLSU dataset was constructed, which included sequences from *Agrocybe*, *Pholiota*, and *Pyrrhulomyces* species (26 type collections). *Cyclocybe* species were selected as outgroups. The sequences used in this study are presented in Table 1.

**Table 1.** Specimen data and DNA sequences analyzed in this study. New DNA sequences are in bold.

Species	Specimen-voucher (Herbarium)	Origin	GenBank accession numbers	
			ITS	nrLSU
*Agrocybe dura*	Ghobad-4299	Iran	MT535724	MT554314
*A. dura*	CBS 246.38	USA	MH855957	MH867453
*A. dura*	CBS 157.63	–	MH858248	MH869851
*A. dura*	Ghobad-4296	Iran	MT535722	MT554312
*A. eduardii*	LE313652 (holotype)	Russia	NR_198215	NG_243087
***A. eduardii***	L24092301 (HMJAU37438)	China, Jilin	**PV839313**	**PV839303**
***A. eduardii***	HMJAU5348	China, Jilin	**PV839314**	**PV839304**
***A. eduardii***	HMJAU23902	China, Jilin	**PV839315**	**PV839305**
***A. eduardii***	HMJAU24841	China, Jilin	**PV839316**	**PV839306**
*A. retigera*	JAUCC2154	China, Zhejiang	MT755839	MN710544
*A. retigera*	FLAS-F-60923	USA	MH211686	MH620258
*A. rivulosa*	CCB 160	USA	KF830098	KF830090
*A. rivulosa*	KUBOT-KRMK-2020-95	India	MW487609	MW485813
*A. vervacti*	GLM 45870	Germany	–	AY207143
*A. vervacti*	G0553	Hungary	–	MK277506
*Cyclocybe cylindracea*	ANGE318	Italy	KM260145	KM260150
*C. cylindracea*	ANGE315	Italy	KM260144	KM260149
*C. parasitica*	voucher BRQ02/24	–	–	AY219580
*Pholiota adiposa*	ET37 (HMJAU37520)	China, Jilin	MN209721	MN251112
*Ph. aurivella*	ET42 (HMJAU37521)	China, Jilin	MN209729	MN251117
*Ph. aurivella*	ET27 (HMJAU37516)	China, Jilin	MN209728	MN251116
*Ph. avellaneifolia*	AHS59589 (TENN-F-028809 isotype)	USA, Idaho	MN209731	MN251120
*Ph. betulicola*	ET31 (HMJAU37328, holotype)	China, Jilin	OP244886	MN251156
*Ph. betulicola*	HMJAU37369	China, Jilin	OP244887	OP223414
*Ph. brunnescens*	PBM3057 (TENN-F-063855)	USA, California	MG735314	–
*Ph. brunnescens*	ET22 (HMJAU37363)	China, Jilin	MN209733	MN251122
*Ph. caespitosa*	TENN-F-015908 (holotype)	USA, Tennessee	NR119908	–
*Ph. carbonaria*	AHS9500 (holotype)	USA, California	MG735288	–
*Ph. conissans*	CBS 243.50	France	MH856603	–
*Ph. cylindrospora*	ET-Ti2 (holotype)	China, Yunnan	PQ013666	PQ013732
*Ph. cylindrospora*	ET-yun2	China, Yunnan	PQ013686	PQ013733
*Ph. decorata*	AHS54770 (TENN-F-028816)	USA, Idaho	MN209734	–
*Ph. ferrugineolutescens*	TENN-F-028807 (isotype)	USA, California	HQ222026	–
*Ph. flavopallid*	ET38 (HMJAU22691)	China, Jilin	MN209737	MN251125
*Ph. fulviconica*	LRH28818 (TENN-F-028818)	USA, Idaho	MN209738	MN251126
*Ph. fulvodisca*	AHS65898 (TENN-F-028820, isotype)	USA, Idaho	MN209739	MN251127
*Ph. gallica*	PRM 933232	France	LN889967	–
*Ph. gallica*	MPU 3478 (holotype)	France	HG007988	–
*Ph. gummosa*	voucher 6610	Italy	JF908581	–
*Ph. highlandensis*	FIRE184 (TENN)	USA, Tennessee	MH348870	–
*Ph. highlandensis*	PBM4085 (TENN-F-071544)	USA, California	MG735288	–
*Ph. humii*	AHS58633 (TENN-F-028822, isotype)	USA, Idaho	MN209740	MN251128
*Ph. lenta*	PBM4233 (TENN-F-074640)	USA, North Carolina	MN209743	MN251131
*Ph. lenta*	ET33 (HMJAU37519)	China, Jilin	MN209742	MN251130
*Ph. limonella*	ET28 (HMJAU37345)	China, Inner Mongolia	MN209747	MN251135
*Ph. luteobadia*	AHS43222 (MICH 11688, holotype)	USA, Michigan	MG735289	–
*Ph. lubrica*	ET4 (HMJAU22678)	USA, Michigan	MN209753	MN251141
*Ph. lubrica*	ET29 (HMJAU37517)	China, Jilin	MN209751	MN251139
*Ph. lurida*	AHS66386 (TENN-F-028770, isotype)	USA, Michigan	MN209757	–
*Ph. marangania*	HLepp856 (CANB574576)	Australia	MG735320	–
*Ph. mixta*	PBM2499 (TENN-F-062357)	USA, Massachusetts	MH016953	–
*Ph. mixta*	PRM 909924	Czech Republic	HG007979	–
*Ph. molesta*	AHS65008 (TENN-F-028830, isotype)	USA, Idaho	MG735296	–
*Ph. molesta*	MTS4953a (WTU 10954)	USA, Washington	MG735309	–
*Ph. multicingulata*	PBM3587 (TENN-F-066655)	Australia	MN209760	–
*Ph. multicingulata*	PDD97861 (TENN-F-063875)	New Zealand	HQ832449	HQ832463
*Ph. multicingulata*	ET23 (HMJAU37414)	China, Yunnan	MN209761	MN251146
*Ph. occidentalis*	AHS58470 (TENN-F-028874, paratype)	USA, Idaho	MN209765	MN251150
*Ph. olivaceophylla*	MICH 290502 (holotype)	USA, California	KF878381	–
*Ph. prolixa*	AHS5027 (TENN-F-028838, isotype)	USA, Michigan	MN209766	–
*Ph. rubronigra*	AHS56192 (TENN-F-028840, isotype)	USA, California	MH016955	–
*Ph. rufodisca*	B925 (TENN-F-028869, paratype)	USA, New Mexico	MN209767	–
***Ph. songjiangensis***	H3tian1 (HMJAU37426)	China, Jilin	**PV839317**	**PV839307**
***Ph. songjiangensis***	H3tian2 (HMJAU37427)	China, Jilin	**PV839318**	**PV839308**
*Ph. spumosa*	ET12 (HMJAU37513)	China, Jilin	MN209776	MN251159
*Ph. spumosa*	ET18 (HMJAU22609)	China, Inner Mongolia	MN209775	MN251158
*Ph. spumosa*	TENN-F-012950	USA, Tennessee	MN149361	–
*Ph. squarrosa*	PBM2735 (TENN-F-062547)	USA, Colorado	DQ494683	DQ470818
*Ph. squarrosa*	AH-48188	Spain	MF345959	–
*Ph. squarrosa*	ET15 (HMJAU37366)	China, Jilin	MN209778	MN251161
*Ph. stratosa*	AHS64684 (TENN-F-028845, isotype)	USA, Michigan	MN209779	–
*Ph. subcaespitosa*	HMJAU 37330 (holotype)	China, Jilin	OP244888	OP223415
*Ph. subsaponacea*	AHS74095 (MICH 5332, holotype)	USA, Idaho	MG735287	–
*Ph. subterrestris*	ET-072240 (holotype)	China, Guizhou	PQ013700	PQ013730
*Ph. subterrestris*	ET-gui2	China, Guizhou	PQ013701	PQ013731
*Ph. terrestris*	JP011(UBC: F19759)	USA, Tennessee	HQ604756	–
*Ph. terrestris*	RAS371 (TENN-F-074807)	USA, Tennessee	MN209781	–
*Ph. velaglutinosa*	AHS9285 (TENN-F-028851, isotype)	USA, Oregon	MH016954	–
*Ph. virgata*	B763 (TENN-F-028832, paratype)	USA, New Mexico	MN209782	–
*Ph. virescentifolia*	TENN-F-020591 (holotype)	USA, Tennessee	NR_119911	–
*Pyrrhulomyces amariceps*	RAS070 (TENN-F-071890)	USA, North Carolina	MG735284	MN251114
*Py. amariceps*	PBM2975 (TENN-F-062733, holotype)	USA, Tennessee	HQ832448	HQ832462
*Py. astragalinus*	AHS60388 (TENN-F-028808)	USA, Idaho	MN149357	–
*Py. astragalinus*	14531	Italy	JF908586	–
*Py. astragalinus*	TFB7720 (TENN-F-054310)	Alaska	MN209722	–
*Py. astragalinus*	MV12616 (TENN-F-061287)	Canada	MN209724	–
*Py. astragalinus*	RHP28708 (TENN-F-028708)	USA, Maine	MN209725	–
*Py. astragalinus*	PBM4330 (TENN-F-074962)	USA, North Carolina	MT187979	MT23744
*Py. astragalinus*	MOS70/345 (TENN-F-037128)	Switzerland	MN149358	–
***Py. astragalinus***	Y083111 (HMJAU37439)	China, Heilongjiang	**PV839321**	**PV839311**
***Py. astragalinus***	G90322 (HMJAU37440)	China, Heilongjiang	**PV839322**	**PV839312**
***Py. pileocystidiatus***	H24091714 (HMJAU37436)	China, Jilin	**PV839319**	**PV839309**
***Py. pileocystidiatus***	64152 (HMJAU37437)	China, Guizhou	**PV839320**	**PV839310**

### ﻿Phylogenetic analysis

Phylogenetic analyses were conducted using maximum likelihood (ML) and Bayesian inference (BI) approaches. A best-fit model of GTR+I+G was selected for the datasets using jModelTest 2 (Guindon and Gascuel 2003; Darriba et al. 2012). The ML analysis with 1000 bootstrap replicates was performed using RAxML 8.2.9 (Stamatakis 2014). Bootstrap support values were calculated to assess the branch confidence. The BI analysis was performed using MrBayes 3.2.7 (Ronquist et al. 2012). Four Markov chains were run for two million generations, with trees and parameters sampled every 1000 generations. The first 25% of the generations were discarded as burn-in, as this coincided with the point at which the average standard deviation of the split frequencies fell below 0.01. The potential scale reduction factors for all the parameters approached 1.0 at the end of the analysis, indicating convergence. The Bayesian posterior probabilities (PPs) were then calculated. The resulting phylogenetic trees from both analyses were visualized using FigTree 1.4.3 (Andrew 2016).

## ﻿Results

### ﻿Phylogeny

**Figure 1.**
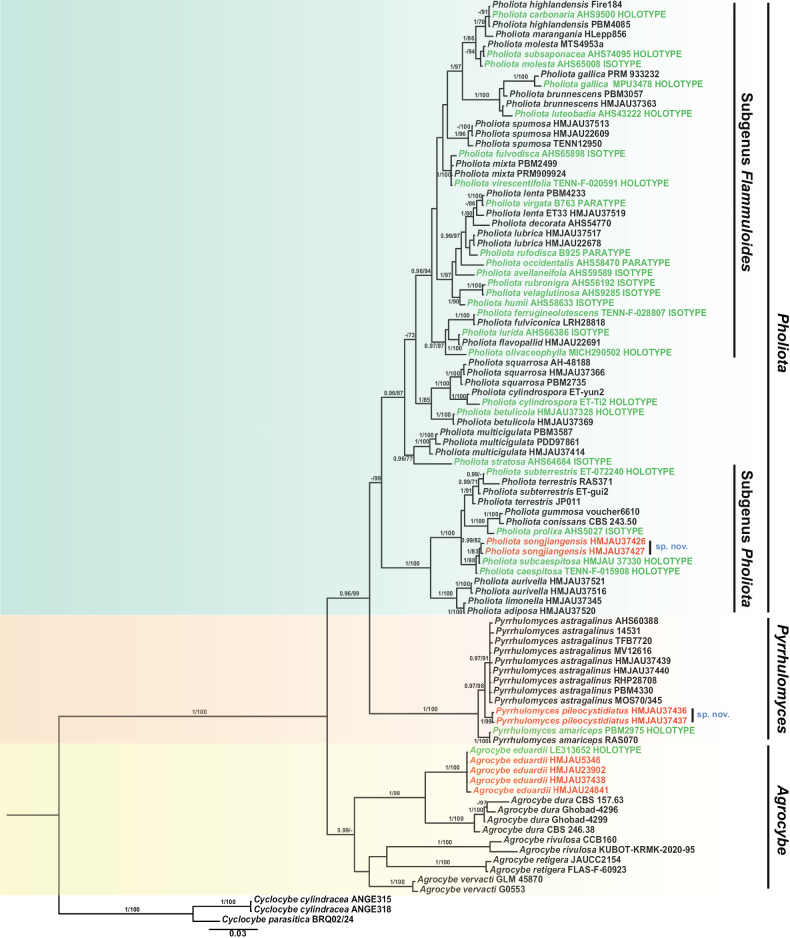
Bayesian inference (BI) phylogram of *Agrocybe*, *Pholiota* and *Pyrrhulomyces* based on dataset (ITS+nrLSU). PPs > 0.95 and bootstrap values > 70% are shown. Types are indicated in green, and the new species and the species newly recorded in China in this study are in red.

In total, 20 sequences (10 ITS and 10 nrLSU) were generated in this study (Table 1), including 8 sequences from new species (4 ITS and 4 nrLSU), and 8 sequences from the newly recorded species (4 ITS and 4 nrLSU). The final general ITS+nrLSU dataset contained 90 ITS and 55 nrLSU sequences and consisted of 2284 characters (with gaps). The phylogram in Fig. 1 displays branch lengths inferred using MrBayes along with support values (PPs and bootstraps) derived from both BI and ML analyses. The topology estimated using ML analysis was nearly identical to that inferred from the BI.

In the phylogram (Fig. 1), three clades were inferred: *Pholiota* (including two major groups: Subgenus Pholiota and Subgenus Flammuloides), *Pyrrhulomyces*, and *Agrocybe*. The two new species described here, *Ph. songjiangensis* and *Py. pileocystidiatus*, respectively clustered within *Pholiota* and *Pyrrhulomyces*, and represented relatively independent lineages. *Pholiota songjiangensis* included two samples and clustered within the group of Subgenus Pholiota as sister to *Ph. caespitosa* A.H. Sm. & Hesler and *Ph. subcaespitosa* E.J. Tian. *Pyrrhulomyces pileocystidiatus* was sister to *Py. astragalinus* and *Py. amariceps*. The species newly recorded in China, *A. eduardii*, and the holotype sample of this species from Russia were gathered together with strong support. This species was sister to *A. dura* (Bolton) Singer with high statistical support.

### ﻿Taxonomy

#### ﻿ Pholiota songjiangensis

Taxon classificationFungiAgaricalesStrophariaceae

E. Tian & C. Lei sp. nov.

74B1D5D0-56D1-5F9B-8B88-F522A443BBDF

860026

Figs 2, 3

##### Etymology.

The epithet refers to the location where the holotype specimens were found.

##### Diagnosis.

Differs from the other *Pholiota* species by its pallid pileus with appressed and concentric squamules; white stipe covered with light yellowish-brown small scales; ellipsoid to ovoid basidiospores with an obvious germ pore; pleurocystidia as chrysocystidia; cheilocystidia with two shapes: elongate-cylindrical with capitulate apex and narrowly lageniform.

##### Holotype.

**China** • Jilin: Jiaohe City, Songjiang County, Shansongling; elev. 520 m, 43°32'48"N, 127°3'8"E, Caespitose, forming fairy rings around decaying tree stumps and on adjacent ground in broadleaf forests, 5 September 2016, Enjing Tian 37426 (holotype: HMJAU!).

##### Description.

**Pileus** 8–35 mm in diam, convex, becoming expanded, with a sticky surface when wet, ground color yellowish white (1A2), sometimes the central part appears wax white to straw yellow (2B3–3B4), decorated with concentric, appressed butter yellow to grayish yellow (4A5–4B5) scales of fibrils agglutinated, the cap margin bears remnants of the veil. **Context** light gray (1C1), sometimes yellowish gray (3C2) where stem meets cap. **Lamellae** attached to slightly running down stem, pallid at first, then cream (4A3) to brownish orange (5C6), medium broad (2–3 mm), L = 44–56, I = 3–7, close, edges even. **Stipe** 10–45 mm long, 3–7 mm wide, central, cylindrical, solid when young but becoming loosely fibrous in age, surface pale gray (1B1) but khaki (4D5) near the base. The veil leaving a distinct annular zone on the stipe; above the annulus smooth, below densely covered with chamois (4C5) scales.

**Figure 2.**
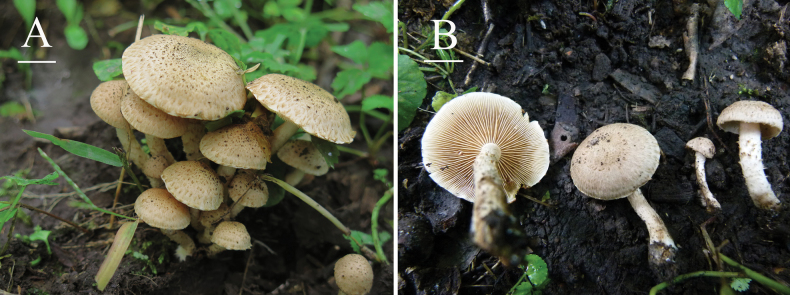
Basidiomata of *Pholiota songjiangensis* A, B. (HMJAU37426, holotype). Photos by Enjing Tian. Scale bars: 1 cm.

**Basidiospores** 5.5–7.5 × 3.5–5 μm, Q = 1.35–1.85, Qm = 1.58 ± 0.25, in face view elliptic to ovate, in side view inequilateral, wall smooth and thick, germ pore obvious, golden brown to linoleum brown (5D7–5E7) in KOH, slightly paler in Melzer’s reagent. **Basidia** 17–22 × 6–7 μm, 2- or 4-spored, clavate, hyaline in KOH. **Pleurocystidia** abundant, as chrysocystidia, 23–74 × 7–10 μm, clavate, clavate with a mucronate apex to subfusiform, with an amorphous highly refractive inclusion, hyaline, chamois (4C5) to oak brown (5D6) in KOH, wall thin and smooth. **Cheilocystidia** 20–56 × 5–7.5 μm, cylindric with a capitate apex to narrowly lageniform, thin-walled, smooth, content homogeneous, hyaline to milk white (1A2) in KOH. **Caulocystidia** not observed. **Gill trama** of parallel hyaline hyphae in KOH and with smooth walls, the cells inflated, up to 20 μm in diam. **Pileipellis** a cutis of oak brown (5D6) to chamois (4C5) hyphae 3–14 μm in diam, thin-walled, smooth to slightly encrusted. **Clamp connections** present in all tissues.

**Figure 3.**
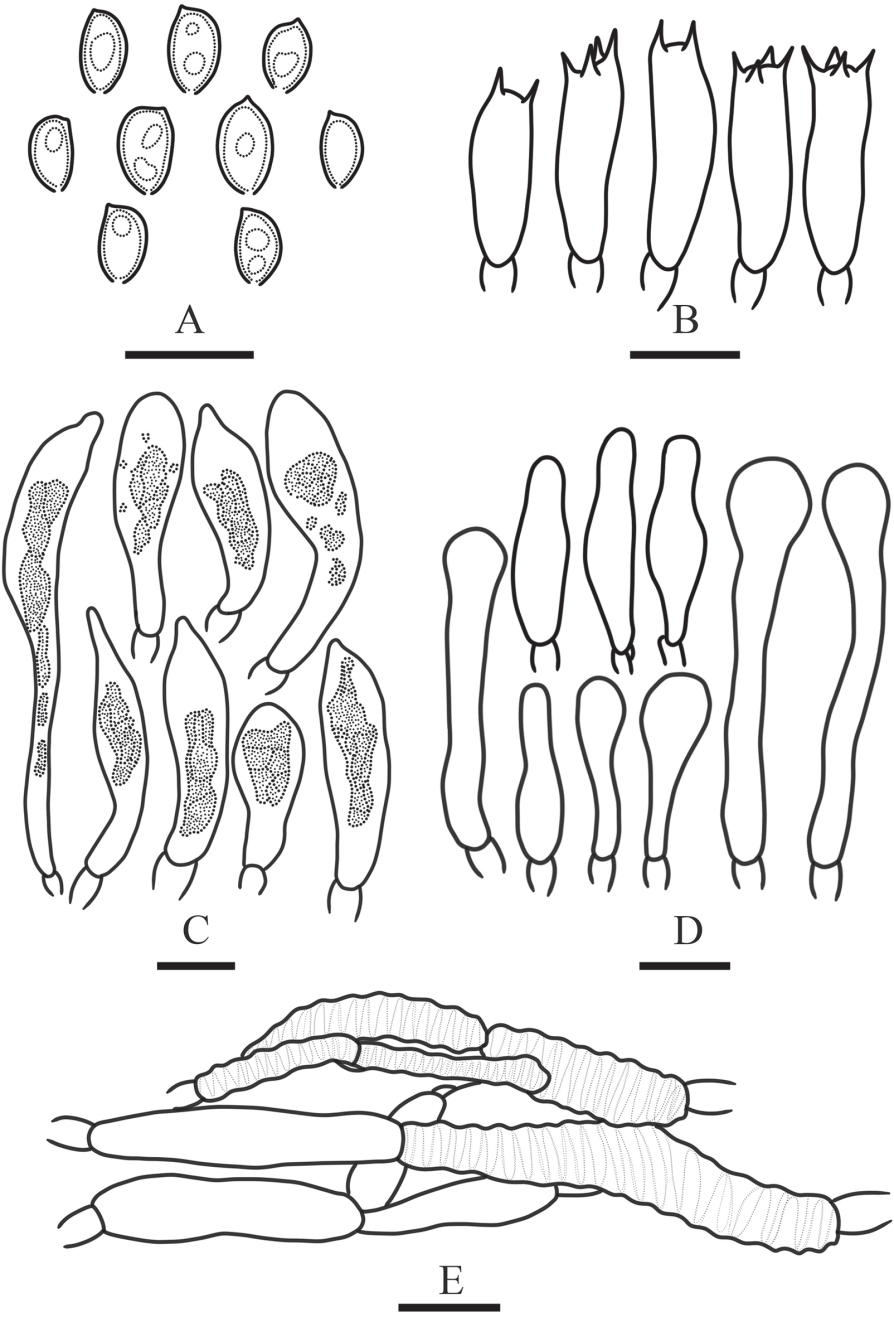
Microcharacters of *Pholiota songjiangensis* (HMJAU37426, holotype). Drawings by Chunyu Lei. A. Basidiospores; B. Basidia; C. Pleurocystidia; D. Cheilocystidia; E. Pileipellis. Scale bars: 10 µm (A–D); 20 µm (E).

##### Habitat.

Caespitose on stumps and/or on soil in broadleaf forests in summer and fall, often forming fairy rings around decaying tree stumps.

##### Additional materials examined.

**China**. • Jilin: Jiaohe City, Songjiang County, Shansongling, clustered at the base of broadleaf tree stumps, 29 August 2020, Hongde Zhai 37427 (HMJAU).

##### Comments.

This species is characterized by a pallid pileus with concentric and appressed butter yellow to wheat scales of agglutinated fibrils, whitish stipe covered with light yellowish-brown small squamules, ellipsoid to ovoid basidiospores with an obvious germ pore, pleurocystidia as chrysocystidia, thin-walled and elongate-cylindrical cheilocystidia with capitulate apex to narrowly lageniform.

*Pholiota songjiangensis* is similar to *Ph. subcaespitosa*, especially in terms of its microcharacteristics. However, the latter has a pileus with an umbonate or depressed disc, covered with brownish fibrillose scales that are appressed and often not apparent in age, and a slimmer stipe (40–70 × 2–5 mm) (Liu et al. 2024), which can be used to distinguish the two species easily. *Pholiota caespitosa* and *Ph. gummosa* (Lasch) Singer are also related to *Ph. songjiangensis*. However, *Ph. caespitosa* lacks cylindrical cheilocytidia and has lamellae with even edges, whereas *Ph. gummosa* has no pleurocystidia in the hymenium (Smith and Hesler 1968).

In the phylogram (Fig. 1), *Ph. songjiangensis* clustered in the Pholiota subgenus Pholiota clade with high statistical support and was sister to *Ph. caespitosa* and *Ph. subcaespitosa* but represented a relatively independent lineage. *Pholiota songjiangensis* differs from *Ph. caespitosa* by ITS (0.8–1%) genetic divergence and from *Ph. subcaespitosa* by ITS (0.3–0.7%) and 28S (2–3 sites) genetic divergence. Therefore, *Ph. songjiangensis* is proposed here as a new Pholiota species belonging to the subgenus Pholiota based on morphological examination and phylogenetic analyses.

#### ﻿ Pyrrhulomyces pileocystidiatus

Taxon classificationFungiAgaricalesStrophariaceae

J. Huang & E. Tian sp. nov.

EB7D2001-00B4-540B-BE29-A5C6B7BA1118

860025

Figs 4, 5

##### Etymology.

Referring to the presence of pileocystidia.

##### Diagnosis.

Characterized by the bright orange-red to ochraceous brown pileus with an obtuse umbo, bitter taste, blackening basidiomata, the pleurocystidia as chrysocystidia, and the presence of broadly clavate and orange-red pileocystidia.

##### Holotype.

**China** • Jilin, Changbai Mountain Ancient Tree Park; 42°01'16"N, 128°03'42"E; on decaying wood in coniferous and mixed forests; 17 September 2024; Jiahui Huang, 37436 (holotype: HMJAU!).

##### Description.

**Pileus** 30–50 mm in diam, hemispherical to obtusely conical, becoming nearly plane but with a broad low umbo, margin undulate, adorned with fibrillose veil remnants, surface viscid when moist, glabrous or with appressed fibrils, ground color champagne (4B4), the central part appears light brown to agate (7E8) when fresh, hygrophanous in appearance, fading to dark gray (1F1) at maturity. **Context** light yellow (4A4), thin to moderately thick. **Lamellae** adnate to sinuate, absinthe yellow (3C5) to caput mortuum (8F7), moderately broad, L = 49–57, I = 1–3, crowded, with paler edges and fimbriate ornamentation. **Stipe** 40–50 mm long, 3–6 mm wide, central, cylindrical, typically curved at base; surface dry, fibrillose or with scattered fibrillose squamules, ash gray (1B2), base brownish orange to feuille morte (5C6–6D7) dark. **Veil** evanescent, dark brown (6F7) to pale (2A2), sometimes forming an arachnoid annulus, stipe hollow.

**Figure 4.**
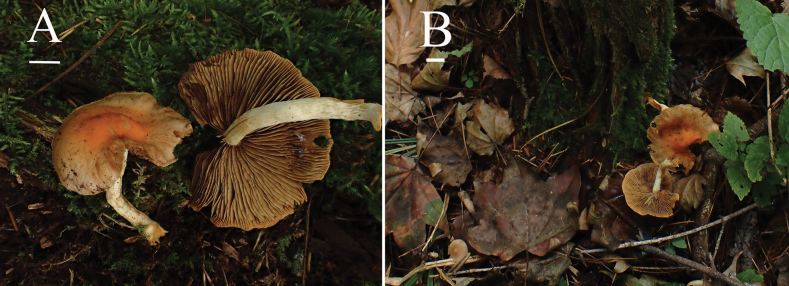
Basidiomata of *Pyrrhulomyces pileocystidiatus* A, B. (HMJAU 37436, holotype). Photos by Jiahui Huang. Scale bars: 1 cm.

**Basidiospores** 6–8 × 4–5 µm, Q = 1.30–1.70, Qm = 1.48 ± 0.10, ellipsoid to ovoid or subovoid in face view, subamygdaliform to inequilateral in side view, wall smooth and slightly thickened, without a germ pore, chamois (4C5) in KOH, paler in Melzer’s reagent. **Basidia** 22–30 × 6–8 µm, 4-spored, narrowly clavate, hyaline to pale yellow (2A3) in KOH. **Pleurocystidia** abundant, as chrysocystidia 38–50 × 10–15 µm, typically clavate or mucronate-clavate, hyaline or light yellow (3A5), containing a refractive amorphous inclusion, thin-walled, smooth. **Cheilocystidia** 40–70 × 5–8 µm, slightly inflated at the base or subbase, with a slender neck (2.5–5 µm wide), apex obtuse to slightly pointed, thin-walled and smooth, hyaline to pale yellow (2A3) in KOH. **Caulocystidia** not observed. **Gill trama** of parallel hyphae hyaline to pastel yellow (2A4) in KOH, subparallel hyphae with inflated cells up to 20 µm in diameter, thin-walled and smooth; subhymenium not gelatinized. **Pileipellis** a ixocutis, hyphae 2.5–5 µm wide, smooth-walled, hyaline or light yellow (3A5). **Pileocystidia** 22.5–42 × 12–18 µm, scattered, clavate, containing small vacuolar inclusions, light orange (5A5) in KOH. **Clamp connections** present in all tissues.

**Figure 5.**
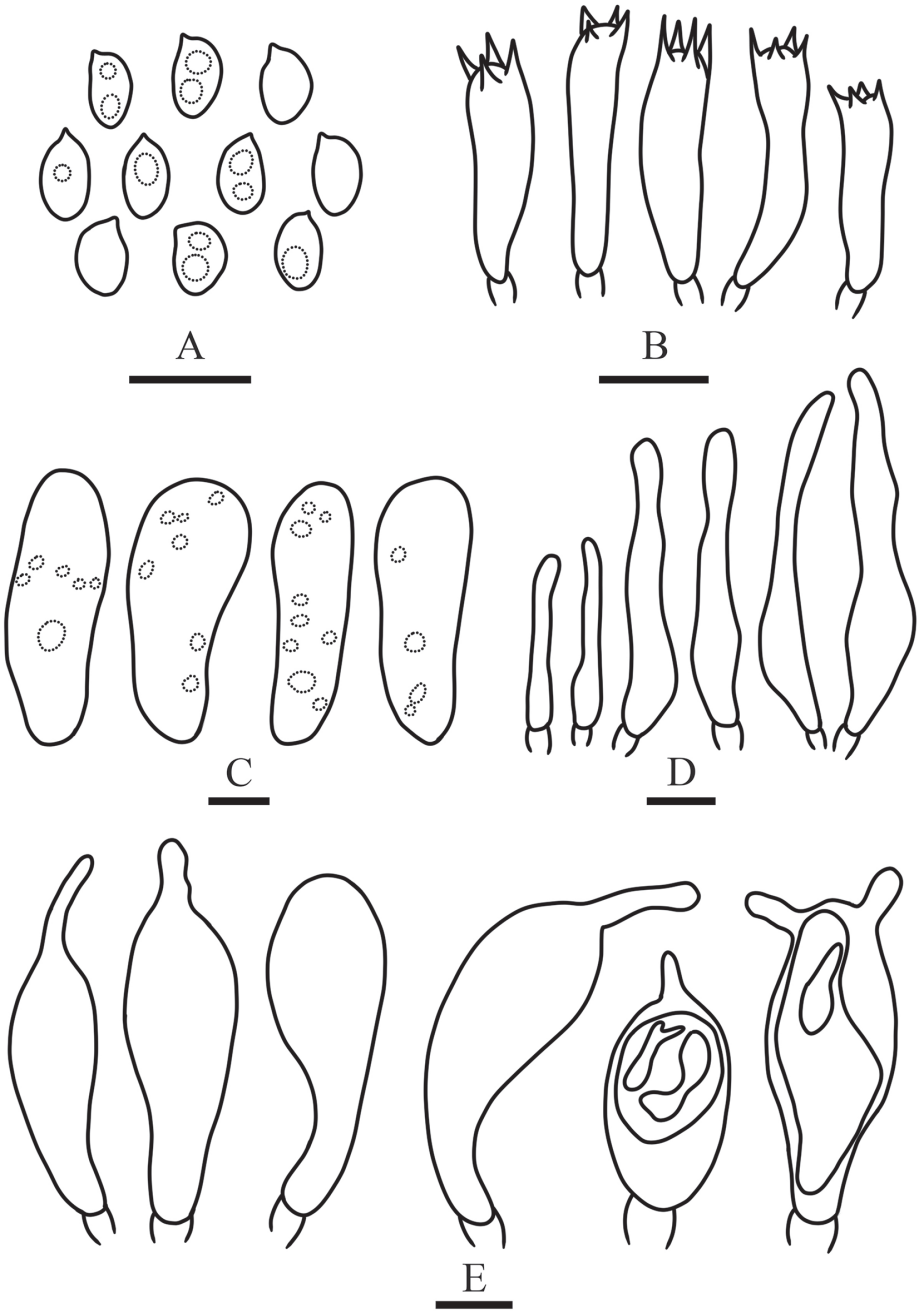
Microcharacters of *Pyrrhulomyces pileocystidiatus* (HMJAU 37436, holotype). Drawings by Chunyu Lei. A. Basidiospores; B. Basidia; C. Pileocystidia; D. Cheilocystidia; E. Pleurocystidia. Scale bars: 10 µm (A–E).

##### Habitat.

Scattered on decaying wood in coniferous or mixed forests, preferring humid environments, in summer and fall.

##### Additional materials examined.

**China** • Guizhou, Bijie City, Nayong County, Shimuzhu Village, on decaying wood in coniferous forest, 21 July 2020, Enjing Tian 37437 (HMJAU).

##### Comments.

This species is characterized by a bright orange-red to ochraceous brown pileus with an obtuse umbo, bitter taste, blackening basidiomata, pleurocystidia as chrysocystidia, and broadly clavate and orange-red pileocystidia.

*Pyrrhulomyces pileocystidiatus* is similar to the only two known species of the genus *Pyrrhulomyces*, *Py. astragalinus* and *Py. amariceps*. *Py. pileocystidiatus* differs from *Py. astragalinus* mainly by the presence of orange-red and clavate pileocystidia, and the latter lacks these special types of end cells of the pileipellis (Smith and Hesler 1968; Tian and Matheny 2021). Furthermore, it is easy to distinguish *Py. pileocystidiatus* from *Py. amariceps* based on the features of pleurocystidia and pileocystidia. The former has only one typically clavate or mucronate-clavate pleurochrysocystidia and orange-red and clavate pileocystidia. In contrast, the latter has two types of pleurocystidia (clavate or mucronate-clavate chrysocystidia with a refractive amorphous inclusion and fusiform-ventricose cystidia with a homogeneous content) and smaller pileocystidia (23–27 × 4–9 µm) with various shapes (Tian and Matheny 2021).

In the phylogenetic analyses, *Py. pileocystidiatus* clustered in the genus *Pyrrhulomyces* clade with high statistical support but represented a relatively independent lineage (Fig. 1). *Pyrrhulomyces pileocystidiatus* differs from *Py. amariceps* by ITS (4–5%) and 28S (4–5 sites) genetic divergence and *Py. astragalinus* by ITS (1–2%) and 28S (0–1 sites) genetic divergence.

Therefore, this species is proposed as a new *Pyrrhulomyces* species based on morphological examination and phylogenetic analyses.

#### ﻿ Agrocybe eduardii

Taxon classificationFungiAgaricalesStrophariaceae

Kiyashko & E. F. Malysheva, Nova Hedwigia 115(1–2): 185 (2022)

DF23F33D-510F-5F19-BCE1-6E36B92C760F

Figs 6, 7

##### Description.

**Pileus** 60–66 mm in diam, initially hemispherical, becoming flattened at maturity, and the margin more or less recurved in age, pastel yellow (2A4) to cognac (6E7) the surface slightly viscid when moist, appearing patchily cracked. **Context** 5 mm thick, milk white (1A2), staining cream to pale yellow (2A3) when bruised, without distinctive odor, and of firm texture. **Lamellae** annexed, of unequal width, initially sunburn (6D5), becoming grayish orange (5B5) at maturity, 2–3 mm broad, L = 44–58, I = 1–4, crowded, edges even. **Stipe** 50–67 mm long, 6–8 mm wide, central, cylindrical to clavate, initially milk white (1A2) and fading to nearly white at maturity, but turning dark brown (7F8) when handled, thickened near the base, straight or slightly curved, and hollow inside.

**Basidiospores** 10–13.6 × 5.4–7.4 μm, Q = 1.6–1.8, Qm = 1.71 ± 0.11, in face view elliptical or oblong, in side view inequilateral, wall smooth and thick, with a distinct germ pore (1–1.4 μm wide) at the apex, golden yellow to brownish yellow (5B7–5C7) in KOH, containing either no oil droplets or multiple oil droplets internally. **Basidia** 25.1–34.7 × 7.8–8.9 µm, 2- or 4-spored, clavate, hyaline in KOH. **Pleurocystidia** 32.2–44.3 × 12.4–17.3 µm, ventriform, generally broadly utriformnon-mucronate, sometimes inflated, often pedicellate, with a short stalk at the base and obtusely rounded at the apex, thin-walled, hyaline in KOH. **Cheilocystidia** abundant, (28.4–)33.1–47.1 × 10.3–16.7 µm, flask-shaped to subclavate, thin-walled, smooth, with a hyaline or straw yellow (3B4) inclusion in KOH. **Caulocystidia** not observed. **Gill trama** of parallel hyphae, 3.7–19 μm in diam, thin-walled, hyaline in KOH. **Pileipellis** a hymeniderm composed of clavate, occasionally coralloid elements 18.2–33.9 × 5.8–12.5 μm, terminal cells thin-walled, hyaline in KOH. **Clamp connections** present in all tissues.

**Figure 6.**
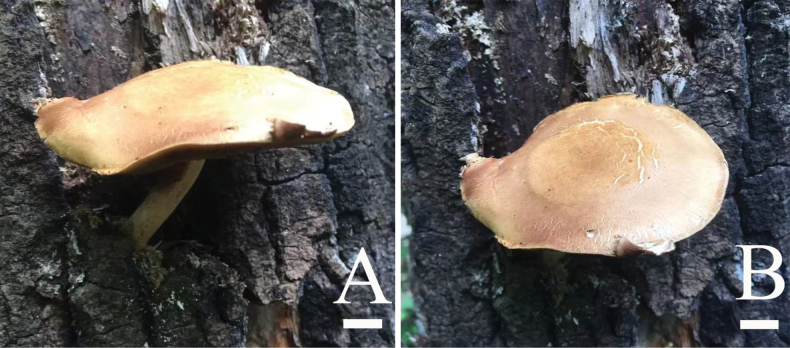
Basidiomata of *Agrocybe eduardii* A, B. (HMJAU 37438). Photos by Bai Wang. Scale bars: 1 cm.

##### Habitat.

Solitary on trunk of broad-leaved trees or on the ground in coniferous and broad-leaved mixed forests in summer and fall.

##### Distribution.

Russia (Kiyashko et al. 2022), China (new distribution).

##### Specimens examined.

**China** • Jilin, Changbai Mountain Nature Reserve, West Mountain, elev. 736 m, 42°24'23"N, 128°28'23"E, solitary on trunk of broad-leaved trees, 24 September 2024, Bai Wang, HMJAU 37438; • Jilin, Baishan City, Jiangyuan County, Sihai Forest Farm, elev. 830 m, 41°56'12.9"N, 126°25'6.6"E, solitary on the ground in coniferous and broad-leaved mixed forests, 11 September 2005, Tolgor Bau, HMJAU5348; • Jilin, Antu County, Erdaobaihe Town, Huangsongpu Forest Farm, solitary on the ground in coniferous and broad-leaved mixed forests, 9 August 2009, Xin Jin, HMJAU 23902; • Jilin, Antu County, Erdaobaihe Town, Huangsongpu Forest Farm, solitary on the ground in coniferous and broad-leaved mixed forests, 9 August 2009, Tolgor Bau, HMJAU 24841.

**Figure 7.**
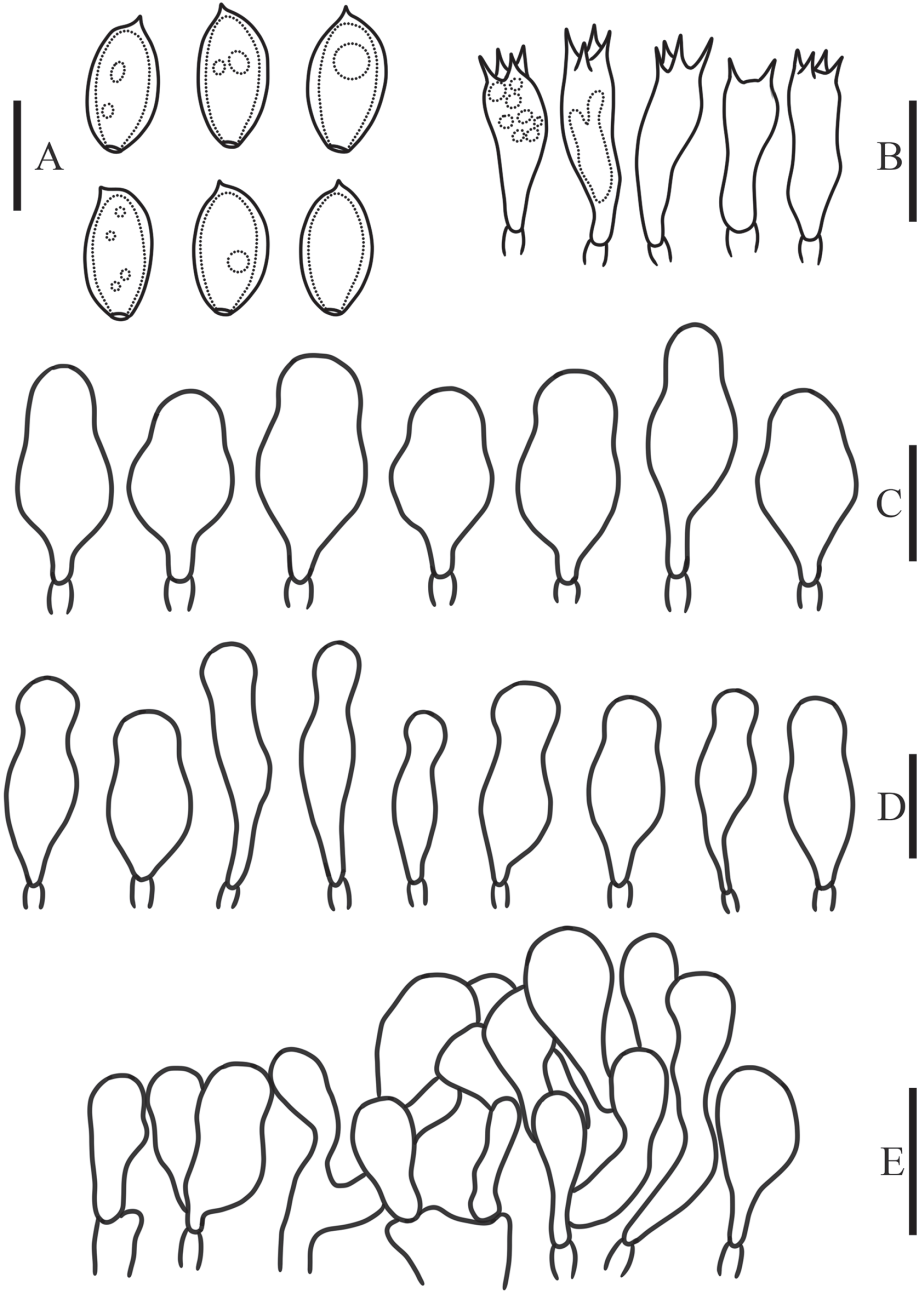
Microcharacters of *Agrocybe eduardii* (HMJAU 37438). Drawings by Chunyu Lei. A. Basidiospores; B. Basidia; C. Pleurocystidia; D. Cheilocystidia; E. Pileipellis. Scale bars: 10 µm (A); 20 µm (B–E).

##### Comments.

*Agrocybe eduardii* from Russia was recently described as a new *Agrocybe* species (Kiyashko et al. 2022). It is characterized by a vividly colored yellow-orange to red-orange pileus, well-developed partial veil persisting as felty patches around the pileus margin, roughly-fibrous to subsquarrose-squamulose stipe with a bulbous base; rather large basidiospores (10–13.6 × 5.4–7.4 μm) with a truncate apex formed by a large germ pore; variable cheilocystidia in shape; and hymeniform pileipellis.

The specimens collected in China in this study were morphologically similar to the original description of *A. eduardii*. In the phylogenetic analyses (Fig. 1), the Chinese samples were grouped with the type of *A. eduardii* monophyletically with high statistical support.

In the phylogram, *A. eduardii* was sister to *A. dura* with high statistical support (Fig. 1). However, morphologically, the former has a vivid orange pileus, margin becoming strongly revolute and lacerate in age, and flask-shaped to subclavate cheilocystidia with a hyaline or straw yellow inclusion in KOH; the latter has a cream-white to pale yellow pileus, short-stipitate vesiculose to broadly ellipsoid or medially constricted elongate-clavate cheilocystidia, and vesiculose to broadly ellipsoid or clavate pleurocystidia with upper-medial constriction (Jin 2012).

## ﻿Discussion

In this study, two new species and one newly recorded species of Strophariaceae from China are described, which contributes to enhance our understanding of the species diversity of Strophariaceae in China and provide basic data for resource utilization of this family.

The subgenus Pholiota has been confirmed to be one of at least two core subgenera in *Pholiota* sensu stricto (Tian and Matheny 2021), mainly characterized by pileus and stipe with distinct scales, and chrysocystidia or very similar sterile cells in the hymenium of many of the species (Smith and Hesler 1968). *Pholiota songjiangensis*, one of the new species described in this study, is affirmed within the subgenus Pholiota based on morphological characteristics and phylogenetic analyses. Recently, several new *Pholiota* species published from China, *Ph. cylindrospora* E.J. Tian, *Ph. subcaespitosa*, and *Ph. subterrestris* E.J. Tian & J.H. Huang, all belong to the subgenus Pholiota (Huang et al. 2024; Liu et al. 2024), which implies that this subgenus possesses abundant species diversity and needs further exploration of its potential resources.

*Pyrrhulomyces pileocystidiatus*, another new species described in this study, is the third species discovered in the genus *Pyrrhulomyces*. The other two known *Pyrrhulomyces* species, *Py. astragalinus* and *Py. amariceps*, are difficult to distinguish from each other morphologically, but can be best separated by genetic divergence at ITS and 28S loci and by the distinct sister-group relationship (Tian and Matheny 2021). However, *Py. pileocystidiatus* can be distinguished from *Py. astragalinus* and *Py. amariceps* not only morphologically but also phylogenetically. At present, *Py. astragalinus* is widely distributed, but *Py. amariceps* has only been found in North America (Tian and Matheny 2021) and *Py. pileocystidiatus* has only been found in China.

*Agrocybe eduardii*, the newly recorded species from China described in this study, grows on wood. Among the species of *Agrocybe*, only a few grow on wood. However, some of them, once belonging to Agrocybe subgenus Aporus, have been transferred to *Cyclocybe*, such as *C. cylindracea* (DC.) Vizzini & Angelini and *C. salicaceicola* (Zhu L. Yang, M. Zang & X.X. Liu) Vizzini (Vizzini et al. 2014). Furthermore, some studies revealed the genus *Agrocybe* remains polyphyletic (Vizzini et al. 2014; Kiyashko et al. 2022; Li et al. 2023), which indicates the further taxonomic revisions of *Agrocybe* are necessary.

### ﻿Key to species of Pholiota subgenus Pholiota from China

**Table:** 

1	Hyphae of the pileipellis non-gelatinized	**2**
–	Hyphae of the pileipellis gelatinized	**5**
2	Annulus present, membranous	**3**
–	Annulus absent, only leaving a fragile annular zone from veil remnants	***Pholiota songjiangensis* sp. nov.**
3	Basidiospores comparatively short (< 6 μm)	***Ph. kodiakensis***
–	Basidiospores comparatively long (> 6 μm)	**4**
4	Cylindrical spores with a minute to inconspicuous germ pore	***Ph. cylindrospora***
–	Ellipsoid spores with a conspicuous germ pore	***Ph. squarrosa***
5	Pileus surface glabrous	***Ph. nameko***
–	Pileus surface covered with appressed squamules	**6**
6	Pileus densely squamulose to fibrillose-scaly, with an evanescent ixocutis	**7**
–	Pileus adorned with concentric rings of pyramidal squamae and a persistent ixotrichoderm	**10**
7	Pileus bright orange-yellow to sulfur yellow or ochraceous; pleurocystidia containing non-refractive contents	**8**
–	Pileus cinnamon-brown to ochraceous russet; pleurocystidia containing refractive contents	**9**
8	Lamellae initially pallid grayish-white; basidiocarps solitary or scattered	***Ph. squarrosoides***
–	Lamellae initially pale clay-brown; basidiocarps caespitose	***Ph. subcaespitosa***
9	Pileus medium-large (2–8 cm broad), lacking orange tones	***Ph. terrestris***
–	Pileus small (1–3.5 cm broad), with orange hues	***Ph. subterrestris***
10	Basidiospores > 5 μm in width	**11**
–	Basidiospores < 5 μm in width	**12**
11	Basidiospores 6–8 μm in width	***Ph. aurivelloides***
–	Basidiospores 4.5–6 μm in width	***Ph. aurivella***
12	Basidiospores distinctly short, length < 6 μm	**13**
–	Basidiospores distinctly long, length > 6 μm	**14**
13	Pileus and stipe lemon-yellow to orange-yellow; stipe adorned with dry, recurved, sulfur yellow squamules	***Ph. flammans***
–	Pileus exhibiting darker coloration; stipe squamules glutinous	***Ph. adiposa***
14	Lamellae initially yellow	**15**
–	Lamellae initially grayish-white to white	**16**
15	A thick and subpersistent annulus present; growing on coniferous wood	***Ph. filamentosa***
–	A annulus absent, only exhibiting fugacious fibrillose annular remnants from the partial veil; growing on deciduous wood	***Ph. squarroso-adiposa***
16	Pileus densely covered with ochraceous-tawny squamae; stipe bearing increasingly dense fibrillose squamules below the annulus	***Ph. abietis***
–	Pileus with scattered pale incarnadine or tawny squamules; stipe bearing sparse recurved squamae	***Ph. limonella***

### ﻿Key to species of *Pyrrhulomyces*

**Table:** 

1	Pileocystidia absent	***Pyrrhulomyces astragalinus***
–	Pileocystidia present	**2**
2	Pleurocystidia of two types: clavate or mucronate-clavate chrysocystidia with a refractive amorphous inclusion and fusiform-ventricose cystidia with a homogeneous content; pileocystidia small (23–27 × 4–9 µm), clavate to fusiform-ventricose, yellowish to yellowish-brown	***Py. amariceps***
–	Pleurocystidia of only one typically clavate or mucronate-clavate chrysocystidia; pileocystidia large (22.5–42 × 12–18 µm), clavate and orange-red	***Py. pileocystidiatus* sp. nov.**

### ﻿Key to species of *Agrocybe* from China

**Table:** 

1	The fruiting body grows on animal dung	***Agrocybe fimicola***
–	The fruiting body grows on grassland, sandy soil, or decaying wood	**2**
2	The mature fruiting body has a cap surface with reticulate cracking	***A. dura***
–	The mature fruiting body has a smooth or wrinkled cap surface	**3**
3	The pileus of the mature fruiting body exhibits pronounced rugose to sulcate features	***A. retigera***
–	The pileus surface of mature fruiting bodies is smooth to slightly rugose	**4**
4	The annulus becomes inconspicuous or absent in aged fruiting bodies	**5**
–	The fruiting body possesses a distinct annulus	**11**
5	Pleurocystidia absent	***A. pediades***
–	Pleurocystidia present	**6**
6	Cheilocystidia hyaline to pale yellow	**7**
–	Cheilocystidia hyaline	**8**
7	Pileus bright orange	***A. eduardii* (new record)**
–	Pileus pale yellow	***A. farinacea***
8	Pleurocystidia with branched apices	***A. arvalis***
–	Pleurocystidia with simple apices	**9**
9	Pleurocystidia and cheilocystidia morphologically similar	***A. striatipes***
–	Pleurocystidia and cheilocystidia morphologically distinct	**10**
10	Basidiospores inamyloid, lacking a distinct germ pore	***A. firma***
–	Basidiospores with a distinct germ pore	***A. putaminum***
11	Basidiospores lacking a germ pore	***A. brunneola***
–	Basidiospores with a distinct germ pore	**12**
12	Pleurocystidia conspicuously large (> 50 μm in length)	**13**
–	Pleurocystidia comparatively small (< 50 μm in length)	***A. praecox***
13	Caulocystidia present	***A. paludosa***
–	Caulocystidia absent	***A. elatella***

## Supplementary Material

XML Treatment for Pholiota songjiangensis

XML Treatment for Pyrrhulomyces pileocystidiatus

XML Treatment for Agrocybe eduardii

## ﻿Additional information

### Use of AI

No use of AI was reported.

### Author ORCIDs

Chunyu Lei https://orcid.org/0009-0001-4275-6813

Guiyin Deng https://orcid.org/0009-0003-2923-2702

Jiahui Huang https://orcid.org/0009-0001-0323-3446

Yalun Shen https://orcid.org/0009-0000-7394-4457

Wenbo Yan https://orcid.org/0009-0006-4262-4024

Enjing Tian https://orcid.org/0000-0001-9742-7569

Yongping Fu https://orcid.org/0009-0007-1118-0843

### Data availability

The research data used in this study can be found in GenBank (https://www.ncbi.nlm.nih.gov) and MycoBank (https://www.mycobank.org).
